# Feasibility of Direct Access Flexible Fibreoptic Sigmoidoscopy for General Practice

**Published:** 1984-07

**Authors:** K. D. Vellacott

**Affiliations:** Surgical Senior Registrar, Bristol Royal Infirmary, Bristol


### Feasibility of Direct Access Flexible Fibreoptic Sigmoidoscopy for General Practitioners

**K. D. Vellacott D.M., F.R.C.S.**
Surgical Senior Registrar, Bristol Royal Infirmary, Bristol

Direct access endoscopy of the upper gastro-intestinal tract is now available in many centres[1,2] with a high diagnostic yield, though it is doubtful whether this has reduced the complications from peptic ulcer disease.[3] This pilot study was established in order to assess the feasibility of running a flexible fibreoptic sigmoidoscopy outpatient clinic with direct access referral for General Practitioners.

### PATIENTS, METHODS AND RESULTS

Fifteen General Practitioners from 3 practices, 2 urban and 1 rural, were invited to refer patients with symptoms of possible colonic disease for flexible sigmoidoscopy. It was explained that early investigation of minor symptoms was to be encouraged for the purpose of the study.

Arrangements were made so that all examinations were performed within 2 weeks of referral. Patients were sent an explanatory letter and on arrival were prepared with a single phosphate enema with no sedation being used for the subsequent examination. A report of the sigmoidoscopy findings along with any recommendations were sent to the referring doctor who was then free to refer the patient to a consultant of his own choice if deemed necessary.

All 15 of the General Practitioners made use of the service referring a total of 48 patients over a period of 9 months. Four patients failed to attend. The mean age of those investigated was 57 years (range 28–85 years). 25 males and 19 females.

The main symptoms for which they were referred were rectal bleeding (29), diarrhoea (10), abdominal pain (4), and tenesmus (1).

The table shows the main diagnosis. A further 10 were deemed to have symptoms due to haemorrhoids and 6 due to probable irritable bowel syndrome. The remaining 15 had normal examinations.

Severe diverticular disease was defined as stricturing or considerable narrowing of the bowel in the presence of multiple diverticula. Where there was doubt a barium enema was advised.

**Table**

| | | |
|---|---|---|
| Carcinoma rectum | 1 | Dukes' B |
| Carcinoma sigmoid | 1 | Dukes' A |
| Adenomatous polyps | 1 | 3 > 1.5 cm diameter |
| Pneumatosis coli | 1 | |
| Severe diverticular disease | 5 | |
| Proctocolitis | 4 | |
| | 13 | (29.5%) |

One patient had multiple large adenomatous polyps and underwent a sigmoid colectomy. Histology showed marked dysplasia in several of them.

### COMMENT

The symptoms of colorectal cancer are non-specific and difficult to distinguish from those of benign colonic or perianal conditions. Patients are often not referred for further investigation unless the symptoms persist for a long time or become severe. In one study 42% of patients, 76% of whom had consulted their family doctor previously with relevant symptoms, were treated as emergencies with complications of colonic cancer.[4] This delay is detrimental to survival as those having elective surgery have an operative mortality of 3.6% compared to 24% for emergency colonic surgery.[5] The average delay after the onset of symptoms until the diagnosis can be as long as 7.5 months, made up of patient, family doctor and hospital delay.[4] Long hospital delays are usually due to poor quality barium enemas or rigid sigmoidoscopies in the presence of faeces. Early flexible sigmoidoscopy would eliminate most of the hospital delays.

Common criticisms of a direct access service are that it would not be possible to sigmoidoscope all patients with symptomatic haemorrhoids and an easy access service might result in General Practitioners being less thorough in their examination of the patient. Colorectal cancer, however, is such a serious problem with less than 10% being diagnosed at an early stage, that only a vigorous attitude towards earlier diagnosis is likely to make any impression on survival. It is particularly encouraging that one patient with a Dukes' A cancer and another with multiple pre-malignant polyps were detected in this small study. As the numbers referred did not provide an excessive work load it is proposed to extend the service to all General Practitioners in Bristol. It remains to be seen whether such a service will prove cost-effective in the long term. Will more early cancers be detected, will request patterns for barium enemas alter and will less patients be admitted as emergencies with complications of colorectal cancer?

I wish to thank Mr. M. Horrocks, Consultant Surgeon for his help in setting up this study, Miss A. Kinghorn for secretarial help, and all the General Practitioners who referred patients.

### REFERENCES

1. GEAR, M. W. L., ORMISTON, M. C., BARNES, R. J., ROCYN-JONES, J. and VOSS, G. C. (1980) Endoscopic studies of dyspepsia in the community: an 'open-access' service. *Br. Med. J.* **280**, 1135.
2. BEAVIS, A. K., BROOY, S. LA., MISIEWICZ, J. J., (1979) Evaluation of one-visit endoscopic clinic for patients with dyspepsia. *Br. Med. J.* **i**, 1387–1389.
3. HOLDSTOCK, G. and COLLEY, S. (1983) Failure of increased use of endoscopy to influence complication rate in peptic ulcer disease. *Br. Med. J.* **287**, 393–394.
4. HOLLIDAY, H. W. and HARDCASTLE, J. D. (1979) Delay in diagnosis and treatment of symptomatic colorectal cancer. Lancet **1**, 309–311.
5. TILL, A. S. (1977) The results of treatment in district general hospitals. Topics in Gastroenterology **5**, 77–85.

